# (Poly)phenol-digested metabolites modulate alpha-synuclein toxicity by regulating proteostasis

**DOI:** 10.1038/s41598-018-25118-z

**Published:** 2018-05-03

**Authors:** Diana Macedo, Carolina Jardim, Inês Figueira, A. Filipa Almeida, Gordon J. McDougall, Derek Stewart, Jose E. Yuste, Francisco A. Tomás-Barberán, Sandra Tenreiro, Tiago F. Outeiro, Cláudia N. Santos

**Affiliations:** 1 Instituto de Tecnologia Química e Biológica António Xavier, Universidade Nova de Lisboa, Av. da República, 2780-157 Oeiras, Portugal; 2 iBET, Instituto de Biologia Experimental e Tecnológica, Apartado 12, 2781-901 Oeiras, Portugal; 3 Instituto de Medicina Molecular João Lobo Antunes, Avenida Professor Egas Moniz, 1649-028 Lisboa, Portugal; 4 Environmental and Biochemical Science Group, The James Hutton Institute, Dundee, DD2 5DA Scotland UK; 5 School of of Engineering and Physical Sciences, Heriot-Watt University, Edinburgh, UK; 6 Research Group on Quality, Safety and Bioactivity of Plant Foods, Department of Food Science and Technology, CEBAS-CSIC, P.O. Box 164, 30100 Campus de Espinardo, Murcia, Spain; 7 CEDOC – Chronic Diseases Research Center, Faculdade de Ciências Médicas, Universidade Nova de Lisboa, Lisboa, Portugal; 8 Department of Experimental Neurodegeneration, Center for Biostructural Imaging of Neurodegeneration, Center for Nanoscale Microscopy and Molecular Physiology of the Brain, University Medical Center Göttingen, Waldweg 33, 37073 Göttingen, Germany; 9 Max Planck Institute for Experimental Medicine, Göttingen, Germany

## Abstract

Parkinson’s disease (PD) is an age-related neurodegenerative disease associated with the misfolding and aggregation of alpha-synuclein (aSyn). The molecular underpinnings of PD are still obscure, but nutrition may play an important role in the prevention, onset, and disease progression. Dietary (poly)phenols revert and prevent age-related cognitive decline and neurodegeneration in model systems. However, only limited attempts were made to evaluate the impact of digestion on the bioactivities of (poly)phenols and determine their mechanisms of action. This constitutes a challenge for the development of (poly)phenol-based nutritional therapies. Here, we subjected (poly)phenols from *Arbutus unedo* to *in vitro* digestion and tested the products in cell models of PD based on the cytotoxicity of aSyn. The (poly)phenol-digested metabolites from *A. unedo* leaves (LPDMs) effectively counteracted aSyn and H_2_O_2_ toxicity in yeast and human cells, improving viability by reducing aSyn aggregation and inducing its clearance. In addition, LPDMs modulated pathways associated with aSyn toxicity, such as oxidative stress, endoplasmic reticulum (ER) stress, mitochondrial impairment, and *SIR2* expression. Overall, LPDMs reduced aSyn toxicity, enhanced the efficiency of ER-associated protein degradation by the proteasome and autophagy, and reduced oxidative stress. In total, our study opens novel avenues for the exploitation of (poly)phenols in nutrition and health.

## Introduction

(Poly)phenols are natural compounds found in a wide variety of foods either as dietary or nutraceutical supplements ^1^. The term polyphenol is used to describe a structure with at least one aromatic ring with one or more hydroxyl groups attached^2^. However, compounds with only one phenolic ring are also referred to as polyphenols, as hydroxycinnamates and phenolic acids. Thus, polyphenols in general, and more accurately, are referred to as (poly)phenols^2^. Epidemiological and clinical studies showed that (poly)phenols can reduce the incidence and prevalence of cardiovascular diseases, cancer, diabetes, inflammation and age-related disorders, as pure (poly)phenols, in extracts or through diet^1–6^. However, for the rational recommendation of (poly)phenols in nutrition, their bioaccessibility and the mechanism of action must be dissected. Thus, the understanding of the influence of digestion on the bioactivities of dietary (poly)phenols is imperative. Studies of digestion in humans and in animal models pose ethical, time and cost concerns. Thus, *in vitro* digestion models to predict phytochemical changes during digestion offer several advantages, including the suitability as a high-throughput approach amenable for mechanistic studies of dietary (poly)phenols in cellular models of disease^7^.

Several studies support the beneficial effects of (poly)phenols and their bioavailability and accumulation even in the brain^8,9^. Strikingly, (poly)phenols can reverse/prevent age-related cognitive decline and neurodegeneration^10^, being attractive as dietary supplements.

Parkinson’s disease (PD) is an age-related neurodegenerative disease for which we still lack effective therapies^11^. The misfolding and aggregation of alpha-synuclein (aSyn) is deeply associated with PD pathogenesis^12^, but the precise underlying molecular mechanisms remain elusive. Among these, oxidative stress and mitochondrial dysfunction are common factors, contributing to disturbances in cellular processes that result in irreversible cell damage and death^13^. In particular, the excessive accumulation of unfolded proteins and oxidative stress that are typical in PD, result in the failure of the endoplasmic reticulum (ER) to cope with the excess of protein load, a process denominated by ER stress^14,15^.

The ER plays several essential functions in the cell as it is responsible for the synthesis of one-third of the total proteome, and is involved in the folding, maturation and posttranslational modification of numerous proteins^14^. In response to ER stress, cells activate the unfolded protein response (UPR)^14–16^, leading to the transcription of genes related with protein homeostasis, including chaperones, and protein degradation and secretion pathways^14^. Sustained ER stress and UPR activation may overwhelm cellular protective mechanisms, ultimately triggering apoptosis^14^. The ER-associated protein degradation (ERAD) is a cellular pathway that targets misfolded proteins for ubiquitination and subsequent degradation by the ubiquitin–proteasome system (UPS) in the cytoplasm^16^. Macroautophagy, hereafter designated as autophagy, is also initiated in response to ER stress caused by misfolded proteins, via ER-activated autophagy (ERAA)^16,17^, which induces a partial UPR and a calcium-mediated signalling cascade. ERAA is a degradation pathway and serves the function of mitigating ER stress and suppressing cell death, as ERAD^16^.

The clearance of soluble aSyn can occur both via the UPS and chaperone-mediated autophagy (CMA)^18^. Under pathological conditions, aSyn inhibits the proteasome and impairs CMA^19,20^, leading to the upregulation of autophagy^21^. On the other hand, the degradation of aggregated aSyn occurs by autophagy^22,23^. Nonetheless, the relationship between autophagy and disease is unclear.

We previously showed that (poly)phenols can protect against aSyn toxicity by promoting its clearance trough autophagy and by modulating aSyn fibrillation^24^. Here, we conducted a pioneering nutritional approach in order to study the bioactivities of (poly)phenol-digested metabolites (PDMs), obtained after *in vitro* gastro-intestinal digestion of edible fruits (FPDMs) and leaves (LPDMs)^25^. We tested (poly)phenols from *A. unedo* (strawberry tree), a native species in regions of Mediterranean climate, since we previously found remarkable protection against oxidative stress, when compared to other (poly)phenol matrices^26^. Additionally, it is known that *A. unedo* (poly)phenols hold potential for the treatment of hypertension^3^, cardiovascular diseases^27^ and cancer^4,28^. Nevertheless, the potential of these (poly)phenols in the context of neurodegeneration has not been explored.

The chemical alterations occurring during *in vitro* digestion were analyzed by LC-MS and the molecular mechanisms of PDMs protection via regulation of autophagy and other targets of aSyn toxicity were analyzed. Interestingly, we found that LPDMs displayed strong protection against aSyn and H_2_O_2_ toxicity in yeast and in human cells, respectively. In addition, these LPDMs reduced aSyn aggregation and induced its clearance through the activation of autophagic and proteasomal function. The LPDMs were able to reduce oxidative stress and aSyn-mediated mitochondrial impairment. We also found that LPDMs regulated transcription of genes involved in the unfolded protein response and Sir2 pathways. In total, our study revealed protective molecular mechanisms of the selected LPDMs in PD models, by modulating the clearance of misfolded proteins and autophagy. Altogether, our study opens novel avenues for the exploitation of nutraceuticals/therapeutics based on the use of (poly)phenols in the context of neurodegenerative diseases.

## Results

### PDMs from *A. unedo* leaves protect against aSyn-induced toxicity

**Figure 1:**
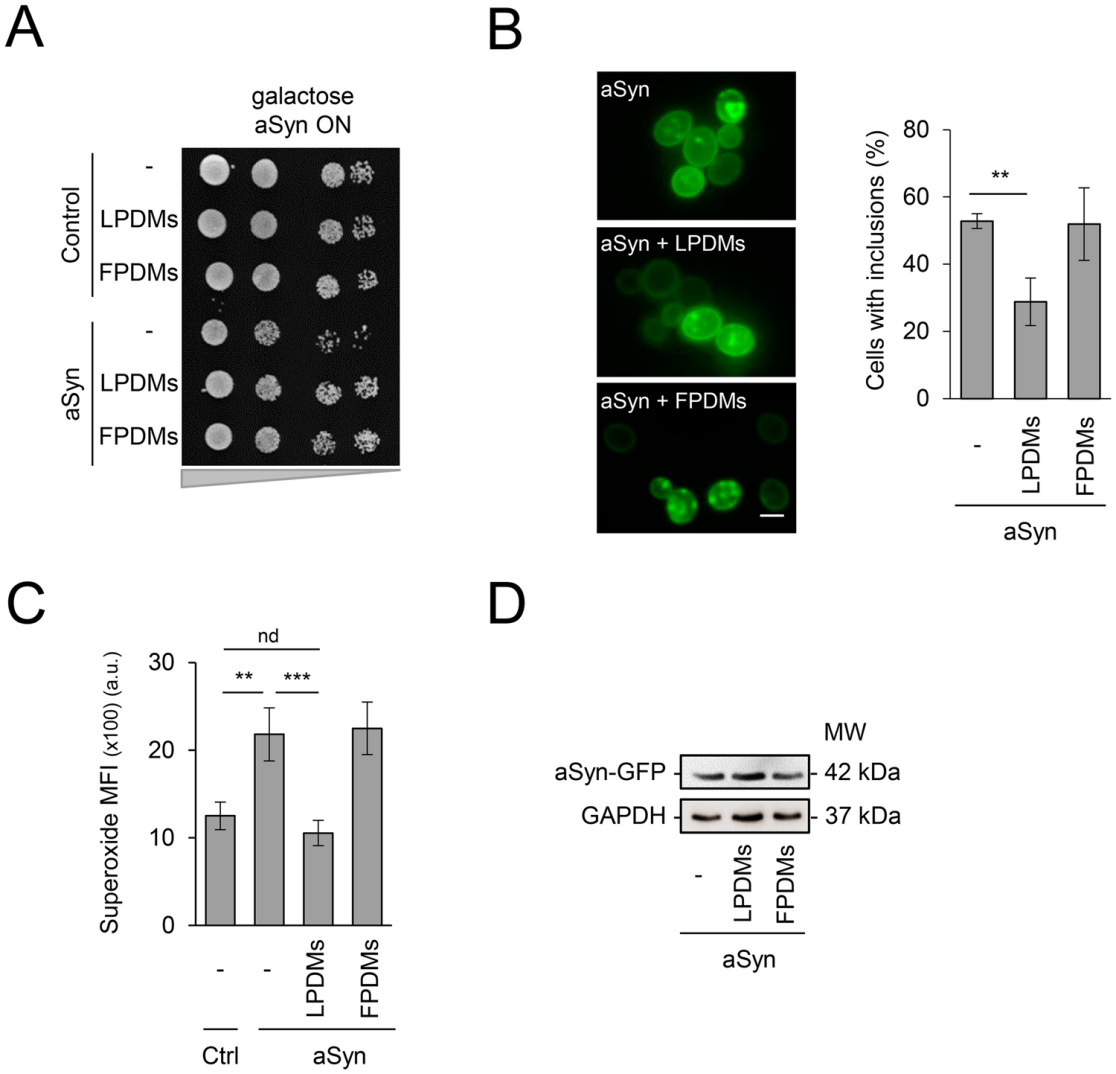
*A. unedo* PDMs reduce aSyn-induced toxicity, inclusion formation and superoxide radical levels. Yeast cells transformed with a plasmid encoding for aSyn-GFP or with the empty vector (control, ctrl), were treated with 62 μg GAE.mL^−1^ of LPDM or FPDMs for 6 h, in galactose liquid medium. (**A**) Cell viability assessed by spot assays, after treatment with PDMs in galactose liquid medium. (**B**) Fluorescence microscopy images of yeast cells (left panel) and respective percentage of cells with aSyn inclusions (right panel). Scale bar: 5 µm. (**C**) Superoxide radical levels evaluated by DHE MFI, assessed by flow cytometry. Results are represented in arbitrary units (a. u.). (**D**) aSyn expression levels assessed by western blot, GAPDH was used as loading control. A representative result is shown, and values represent the mean ± SD of three independent experiments. Statistically significant differences between the indicated treatments are shown, **p < 0.01, ***p < 0.001 or nd as not different for p < 0.05.

We used an established model of PD based on the expression of human aSyn in yeast cells^19,29^ to dissect the mechanisms of protection by *A. unedo* PDMs. The concentration of 62 µg GAE.mL^−1^ was selected as non-toxic based on viability assays (Fig. S1). Yeast cells grown in raffinose medium were transferred to galactose medium, to induce aSyn expression, and treated at the same time with PDMs for 6 h. After this treatment, cellular viability was assessed by spot assays (Fig. 1A). Importantly, we observed that leaf (LPDMs) or fruit (FPDMs), had no effect on the control cells growth. Remarkably, PDMs were able to suppress toxicity induced by aSyn expression, while cells treated with LPDMs grew similarly to control cells (Fig. 1A). Protection was also observed by the reduction of PI positive cells assessed by flow-cytometry when aSyn expressing cells were treated with LPDMs (Fig. S2). Both LPDMs and FPDMs had a strong protective effect on growth (spot tests, Fig. 1A). However, the effect on membrane integrity was not as strong (PI, Fig. S2).

aSyn toxicity in yeast cells is associated with the formation of cytosolic inclusions^19^. Thus, we then assessed if the enhancement in cell viability was accompanied by alterations in the inclusion formation of aSyn, by fluorescence microscopy. Interestingly, treatment with LPDMs significantly reduced the percentage of cells displaying aSyn inclusions (Fig. 1B). However, the treatment with FPDMs had no effect on inclusions formation (Fig. 1B).

Oxidative stress is another central event in PD being a known trigger of aSyn misfolding and aggregation^30^. Conversely, aSyn aggregation induces mitochondrial dysfunction and ROS production^31^. Accordingly, we then analyzed the effect of PDMs treatment on ROS levels in yeast cells expressing aSyn. Superoxide radical levels were evaluated by flow cytometry (FCM), using dihydroethidium (DHE), which is an indicator of mitochondrial metabolic function^32^, a known target of aSyn-mediated toxicity^33^. As observed before, aSyn expression led to an increase in superoxide production (Fig. 1C)^24^. Treatment with LPDMs substantially reduced the levels of superoxide in cells expressing aSyn (Fig. 1C).

We next assessed whether the differences in toxicity and inclusion formation were due to different expression levels of aSyn induced by the PDMs treatments. Using western blot analyses, we found that the PDMs did not affect aSyn protein expression levels (Fig. 1D).

Overall, these results suggest that LPDMs is cytoprotective in a yeast model of PD by reducing inclusion formation and improving redox homeostasis. For this reason, we continued our study focusing on LPDMs and the chemical profile of its (poly)phenols was evaluated.

### Chemical characterization of leaf fractions

LC-MS analysis of the *A. unedo* leaf extracts before (original) and after (LPDMs) digestion (Table 1) confirmed that they were mainly composed of (poly)phenols previously described as having bioactivities^2,3,8,24^. The chemical composition of the LPDMs used has been partially previously described^26^. The major class represented was the flavonols, with quercetin, myricetin, and kaempferol glycosides present (Table 1). As described flavonols are relatively stable to digestion^25^. The galloylated flavonols and the flavanols, epigallocatechin and epicatechin, were less stable and their levels were reduced in the LPDMs. Therefore, the main effect of digestion was the concentration of certain components, and removal of other less stable components from the digested samples. No significant breakdown products were noted, although it is possible that the apparent increase in certain flavonol glycosides was influenced by the drop in galloylated components.

**Table 1:** (Poly)phenol metabolites present in *A*. *unedo* leaf before (original) and after in vitro digestion (LPDMs). (Poly)phenol putative identifications, structure and classes are presented, as well as MS (m/z and MS2) and LC (retention time and PDA) data.

Class	Compound	MS Data		LC Data		Predicted Formula	Relative variation
		M/Z	MS^2^	RT	UV_max_		
		[M − H]^−^		(min)	(nm)	[M-H]	
Flavanols	Epigallocatechin	305	261, 179	1.65	285	C_15_H_13_O_7_	↓
	Epicatechin (EC)	**289** (335)	**245**, 205,179	3.48	280	C_15_H_13_O_6_	↓
	EC dimer	577	423, **289**	3.49	275	C_30_H_25_O_12_	↓
Flavonols	Myricetin-galloyl-hexoside	631	479, **317**	5.87	350, 285	C_29_H_27_O_16_	↓
	Myricetin hexoside	479	317	6.63	350	C_21_H_19_O_13_	↓
	Quercetin-galloyl-hexoside	615	463, 301	7.32, 7.59	350, 285	C_29_H_27_O_15_	↓
	Myricetin rhamnoside	463	**317**	7.75	350	C_21_H_19_O_12_	↓
	Myricetin rhamnoside	463	**317**	8.01, 8.30	350	C_21_H_19_O_12_	↓
	Quercetin pentoside (arabinoside)	433	**301**	8.99	350	C_20_H_17_O_11_	↑
	Quercetin rhamnoside	447	**301**	9.24	350	C_21_H_19_O_11_	↑
	Kaempferol pentoside	417	**285**	9.75	350	C_20_H_17_O_10_	↑
	Kaempferol rhamnoside	431	**285**	10.01	350	C_21_H_19_O_10_	↑
Ellagitannins	Strictinin	633	463, **301**	5.26	280	C_27_H_22_O_18_	↓
Phenolic acids	Hydroxybenzoic acid glucoside	**299** (345)	137	2.52	270	C_13_H_15_O_8_	↑
	Arbutin gallic acid ester	423	355, 313, **261**	3.72	285	C_19_H_19_O_11_	↑
	Digalloyl shikimic acid	477	**325**, 169	4.71	280	C_21_H_17_O_13_	=
	Hydroxybenzoyl arbutin	**391** (437)	**281**, 137	7.25	285	C_19_H_19_O_9_	↑
	Roseoside	**385** (431)	309, 179	5.18	285	C_19_H_29_O_8_	↑
	Rehmaionoside C	433*	387, 301	6.86	280	C_20_H_33_O_10_	↑

The *m/z* values in bold are the major ions or fragments. Values in brackets are formic acid adducts. All components were noted in the original extract and their fate in the digest sample is noted in the last column (LPDMs *vs*. Original). The formulae were predicted from exact mass data with ppm of <2 ppm in all cases.

*This is tentatively identified as the formic acid adduct of *m/z* 387, and that has a molecular formula (METLIN) of C_19_H_31_O_8_ with structure such as Rehmaionoside C, which is a glycosidic terpene that will show UV absorption at 280 nm but with no absorption at 350 nm.

The component with *m/z* 423 was increased by digestion and was subjected to further analysis by QTOF-NMR for identification. The mass and formula are consistent with a gallic acid ester of arbutin, which naturally occurs in three different structural combinations. The ^1^H NMR showed signals consistent with aromatic groups corresponding of hydroquinone, gallic acid and also the sugar moiety (Table 1, Fig. S3). Additionally, three other compounds were increased after digestion (Table 1) and initial MS data suggests that these may be roseoside, a megastigmane glucoside and rehmaionoside C a monoterpenoid with potential bioactivity^34,35^ and hydroxybenzoyl arbutin previously reported in *A. unedo*^36^. However further analysis is required to confirm these identifications.

### LPDMs modulate the oligomerization of aSyn

**Figure 2:**
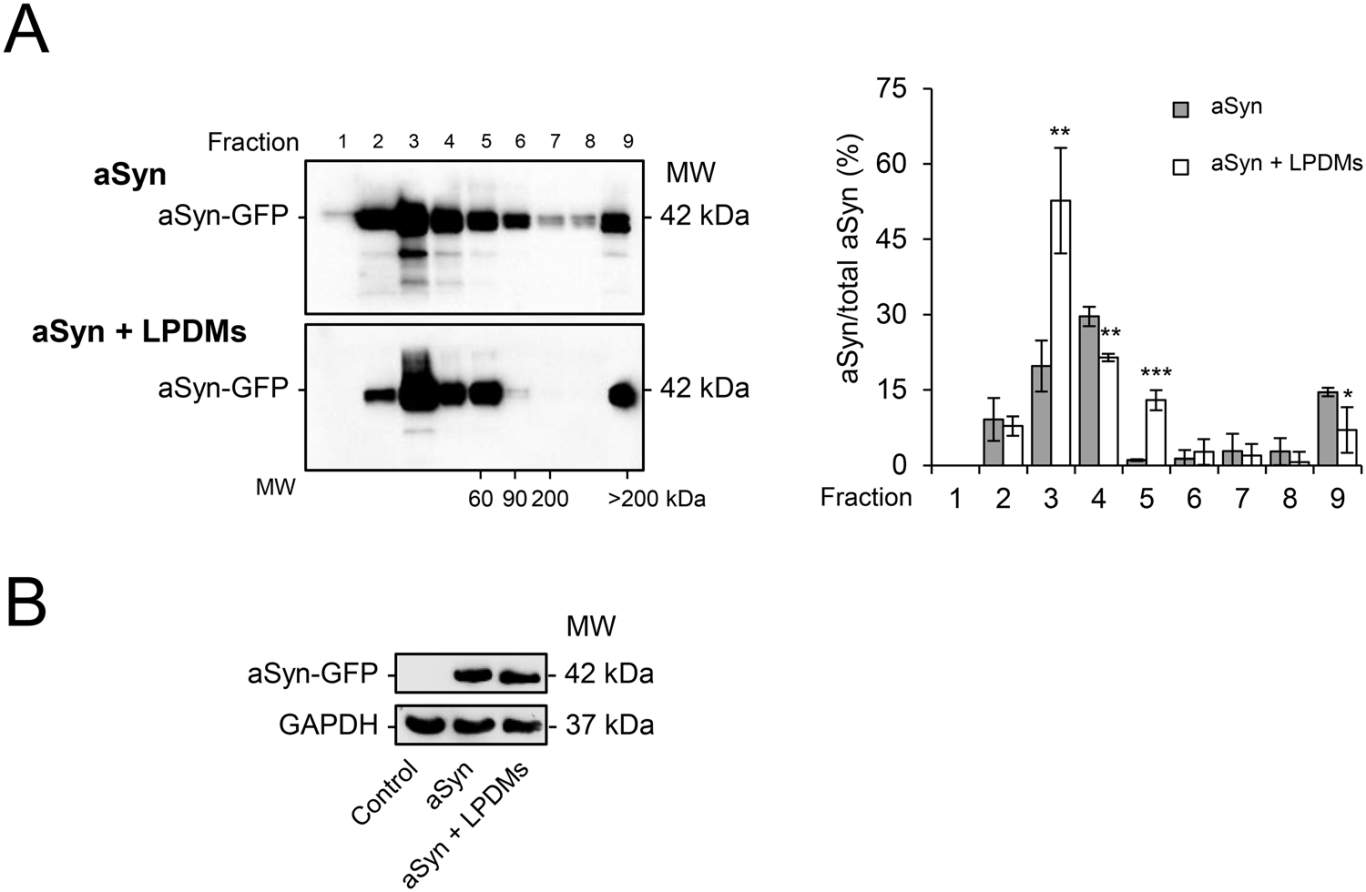
*A. unedo* LPDMs modulate the biochemical nature of aSyn oligomeric species. Yeast cells were treated with 62 μg GAE.mL^−1^ of LPDMs for 6 h, in galactose medium. (**A**) Oligomeric aSyn species resolved using sucrose gradients. The resulting fractions were separated on an SDS-PAGE gel followed by immunoblotting for aSyn (left panel); quantification of aSyn in each fraction normalized by total aSyn (right panel). (**B**) The samples were submitted to the sucrose gradients and evaluated for aSyn expression levels assessed by immunoblot. A representative result is shown, and values represent the mean ± SD of three independent experiments. Significantly different results are indicated between “aSyn” and “aSyn + PDMs” treatment for each fraction. Statistically significant differences between the indicated treatments are shown, *p < 0.05, **p < 0.01, ***p < 0.001.

In order to determine the effect of LPDMs on the biochemical nature of the aSyn species formed in yeast cells, we performed centrifugation in sucrose gradients, as previously described^37^. Briefly, protein fractions corresponding to different molecular weights (numbered from 1 to 9 in the Fig. 2A) were collected from the sucrose gradient and analyzed by SDS-PAGE and immunoblots. The amount of aSyn in each fraction was then evaluated by densitometry (Fig. 2A). aSyn oligomeric species were found dispersed throughout the various sucrose gradient fractions (Fig. 2A). Interestingly, upon treatment with LPDMs, we found that fraction 3 was enriched in aSyn species, indicating the presence of smaller molecular weight species, while the levels of aSyn in fraction 9 (oligomers with molecular weights larger than 200 kDa) were reduced (Fig. 2A, right panel). We confirmed that these changes were not due to differences in the loading of the total protein using GAPDH as a loading control (Fig. 2B). Thus, we concluded that LPDMs modulated the nature of aSyn species formed in yeast cells, reducing the formation of higher molecular weight oligomeric species of aSyn, which are potentially more toxic^12^.

### LPDMs promote the clearance of aSyn by restoring proteasomal and autophagic activities

**Figure 3:**
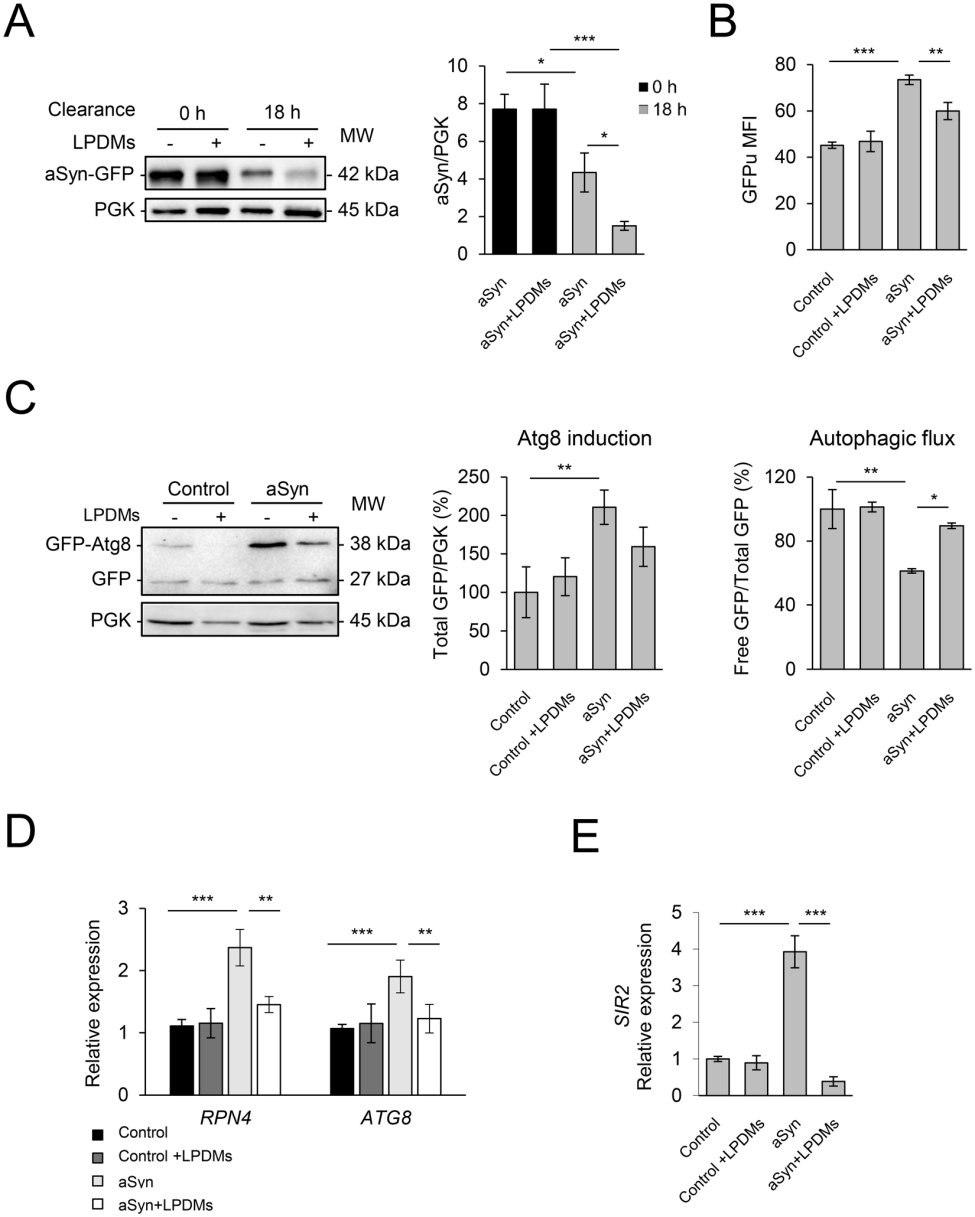
*A. unedo* LPDMs increase the clearance of aSyn by promoting autophagy and proteasomal function. Yeast cells were treated with 62 μg GAE.mL^−1^ of LPDMs for 6 h, in galactose medium. (**A**) Clearance of aSyn evaluated by western blot at 6 h of aSyn induction (0 h clearance) and after18 h in glucose medium (18 h of clearance) (left panel), and respective quantification normalized to PGK levels used as loading control. (**B**) Proteasome impairment evaluated by flow cytometry (FCM) using GFPu median fluorescence intensity (MFI), after 6 h of aSyn induction. (**C**) Autophagy evaluated by GFP-Atg8 processing assay assessed by western blot (left panel), after 6 h of aSyn induction. Atg8 induction quantified by the total GFP signal (GFP-Atg8 and free GFP signal, detected with anti-GFP) (middle panel); autophagic flux quantified by measuring the vacuolar degradation of the Atg8 domain reporter (ratio of free GFP to total GFP signal) (right panel). (**D**) *RPN4* and *ATG8* gene relative expression levels assessed by qRT-PCR. (**E**) *SIR2* relative expression levels assessed by qRT-PCR. A representative result is shown, and values represent the mean ± SD of at least three independent experiments. Statistically significant differences between the indicated treatments are shown, *p < 0.05, **p < 0.01, ***p < 0.001. Full-length immunoblots are shown in Supplementary Figure S4.

The impairment of protein clearance pathways is implicated in the pathogenesis of PD, leading to the accumulation of misfolded and aggregated aSyn^12,38^. Therefore, we next evaluated the effect of LPDMs treatment on aSyn clearance (Fig. 3A). Briefly, 6 h after induction of aSyn expression in galactose medium in the presence of LPDMs (0 h of clearance), cells were switched to glucose-containing medium in order to repress aSyn expression. Cells were then grown in glucose medium for 18 h (18 h clearance), the degradation/clearance of aSyn was assessed by western blotting. Cells treated with LPDMs during the induction period presented lower levels of aSyn after 18 h of clearance (Fig. 3A), suggesting that LPDMs promoted the degradation of aSyn. Thus, we then analyzed the effect of LPDMs on proteostasis pathways.

The clearance of aSyn has been associated with both UPS and autophagy-lysosome pathway^18^. The effect of LPDMs on proteasome activity was analyzed using an unstable GFP reporter (GFPu) consisting of GFP fused with a constitutive degradation signal (CL-1) that promotes its rapid degradation by the UPS^19^. FCM analysis revealed that, upon aSyn expression, the levels of GFPu considerably increased (Fig. 3B) indicating that aSyn impaired proteasome function, as previously reported^19,24^. Interestingly, cells expressing aSyn and treated with LPDMs presented reduced levels of GFPu, indicating an attenuation of the proteasome impairment (Fig. 3B). Treatment with LPDMs had no effect on the levels of GFPu in control cells not expressing aSyn (Fig. 3B), suggesting that this effect was specific for cells expressing aSyn.

Subsequently, we tested whether autophagy was affected by LPDMs using the GFP-Atg8 processing assay as a reporter^39,40^. Atg8 is one of the key molecules involved in autophagy and its conjugation to the autophagosomal membrane, through an ubiquitin-like conjugation system, is essential for autophagy in eukaryotes^40^. We observed that aSyn expression led to a significant increase in Atg8 induction and reduced autophagic flux (Fig. 3C), suggesting that aSyn interfered in autophagy function as previously described^24^. Remarkably, when aSyn-expressing cells were treated with LPDMs the autophagic flux was restored to control levels (Fig. 3C). However, in the control cells not expressing aSyn, both Atg8 induction and autophagic flux remained unchanged when cells were treated with LPDMs (Fig. 3C).

To reinforce the findings described above, we then investigated the involvement of the proteasome and autophagy pathways, by evaluating the mRNA levels of *RPN4* and *ATG8* genes, since their transcriptional regulation is well characterized. *RPN4* encodes for a transcription factor that regulates the expression of proteasome genes^41^, and is upregulated by the UPR^42^. We observed that *RPN4* transcript levels increased upon aSyn expression (Fig. 3D). In contrast, in cells treated with LPDMs the *RPN4* transcript levels did not increase (Fig. 3D). Similarly, aSyn expression induced *ATG8* expression, as expected, considering the Atg8 induction observed in the GFP-Atg8 processing assay (Fig. 3C). However, upon LPDMs treatment, *ATG8* transcript levels were similar to those in control cells not expressing aSyn (Fig. 3D).

To gain further insight into the regulation of proteostasis by LPDMs treatment, we then analyzed a known inducer of autophagy and modulator of aSyn toxicity, the Sir2^43^. Sir2 is a deacetylase of the sirtuin family and functions as a regulator of autophagy and mitophagy^44^. Upregulation of Sir2 is linked with the induction of excessive autophagy and a resulting increase in aSyn toxicity, in aged yeast cells^43^. Consistently, we observed that aSyn expression increased the transcript levels of *SIR2* under the conditions tested (Fig. 3E). However, upon treatment with LPDMs, the *SIR2* expression levels were similar to the control levels (Fig. 3E). Altogether, these results indicate that the LPDMs might play an important role in regulating autophagy, and that one of the mechanisms involved might be through Sir2 modulation.

### LPDMs modulate the unfolded protein response and mitochondrial function

**Figure 4:**
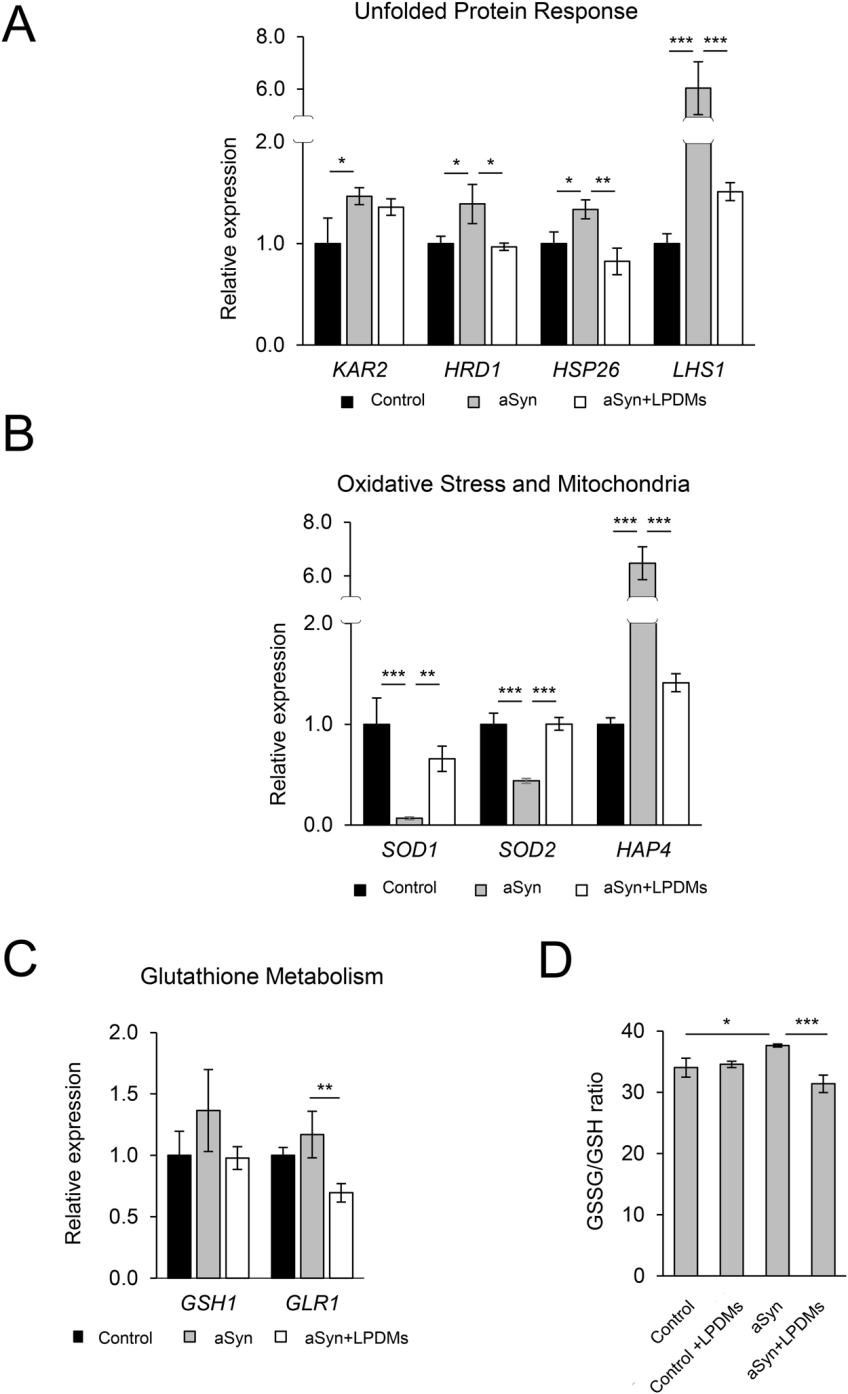
Effect of *A. unedo* LPDMs in the unfolded protein response and oxidative stress. Yeast cells were treated with 62 μg GAE.mL^−1^ of LPDMs for 6 h, in galactose medium. (**A**) Transcript expression levels of genes involved in the unfolded protein response, assessed by qRT-PCR. (**B**) Transcript expression levels of genes involved in oxidative stress response (*SOD1*, *SOD2*) and mitochondrial biogenesis (*HAP4*), assessed by qRT-PCR. (**C**) Transcript expression levels of genes involved in glutathione metabolism, assessed by qRT-PCR. (**D**) GSSG/GSH ratio, GSSG and GSH levels assessed by HPLC. Values represent the mean ± SD of three independent experiments. Statistically significant differences between the indicated treatments are shown, *p < 0.05, **p < 0.01, ***p < 0.001.

Due to the observed effect of LPDMs on the clearance of aSyn, we then evaluated the effect of LPDMs treatment on the expression levels of a set of UPR related genes. We assessed the mRNA levels of *KAR2*, *LHS*1*, HSP26* and *HRD1*. Here, we observed that aSyn expression induced an increase in the expression levels of all the UPR related genes evaluated (Fig. 4A). On the other hand, LPDMs treatment reduced the levels of *LHS1*, *HRD1* and *HSP26* transcripts (Fig. 4A), suggesting that LPDMs were able to attenuate the UPR and the associated ER stress.

Oxidative stress and mitochondrial dysfunction are known triggers of protein misfolding and ultimately result in ER stress. *SOD1*, *SOD2* and *HAP4* mRNA levels were analysed to gain additional insight into the effect of LPDMs treatment on mitochondrial function (Fig. 4B). Sod1 and Sod2 are superoxide dismutases responsible for detoxifying superoxide radical^45^. Hap4 is a global regulator of mitochondrial electron transport and activates their expression^46^. aSyn expressing cells displayed lower levels of *SOD1* and *SOD2* and, remarkably, higher levels of *HAP4*, in comparison to the control cells (Fig. 4B). Notably, upon treatment with LPDMs, *SOD1* and *SOD2* and *HAP4* levels were similar to the control cells. For *HAP4* its levels in aSyn expressing cells were drastically reduced by LPDMs (Fig. 4B).

We then evaluated the major low-molecular-weight redox buffer, glutathione (GSH), a tripeptide that plays a pivotal role in the protection against oxidative damage, detoxification from xenobiotics and endogenous toxic metabolites^47^. We assessed both the levels of the key enzymes involved in GSH synthesis and also the ratio of oxidized glutathione to reduced glutathione (GSSG/GSH). Interestingly, the levels of *GSH1* and *GLR1* were not significantly affected by aSyn expression. However, we observed a slight but significant reduction in *GLR1* mRNA levels in cells expressing aSyn treated with LPDMs (Fig. 4C). The cellular glutathione pools were also determined by HPLC, since the GSSG/GSH ratio is a measure of the cell redox state^47^. We observed that aSyn expression increased the GSSG/GSH ratio, suggesting an increase in oxidative stress. Treatment with LPDMs reduced this ratio, suggesting a reduction of oxidative stress (Fig. 4D).

### LPDMs modulate aSyn aggregation, protect from oxidative stress and promote mitochondrial function in human cells

**Figure 5:**
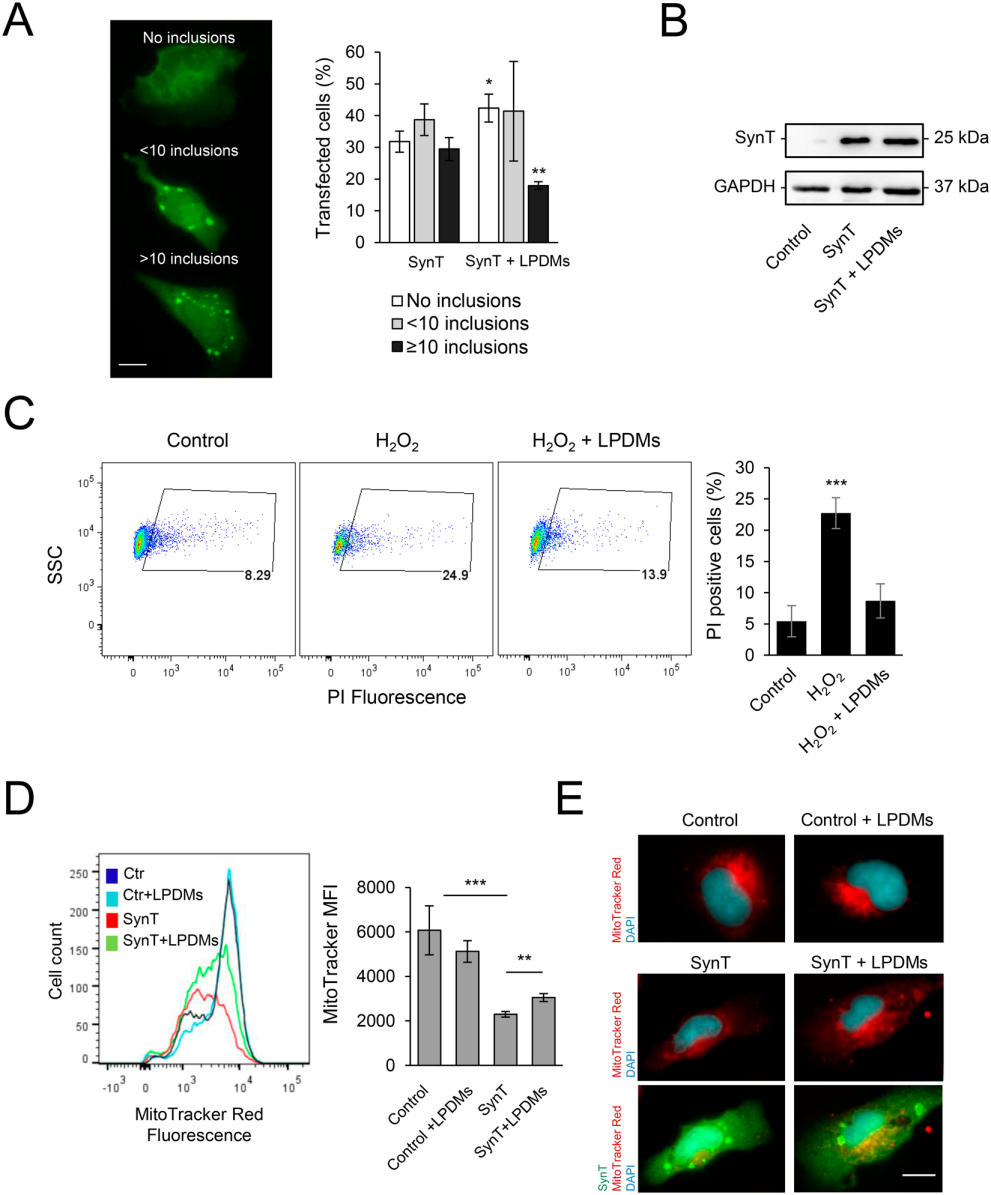
*A. unedo* LPDMs modify the distribution of aSyn inclusions, protect from H_2_O_2_-induced toxicity and promote mitochondrial function in H4 cells. (**A**) H4 human neuroglioma cells were transfected with SynT (SynT and Synphilin-1) for 24 h and treated with 2 µg GAE.mL^−1^ LPDMs for 6 h. Subsequently, cells were subjected to immunofluorescence labeling and the percentage of cells with inclusions was determined by fluorescence microscopy (left panel). Cells were classified by cells without inclusions (□), with less than 10 inclusions (), or with 10 or more inclusions (■) (right panel). Scale bar: 10 µm. (**B**) SynT levels of H4 cells transfected with the empty vector (control), co-transfected with SynT and Synphilin-1 (SynT) or treated with LPDMs (SynT + LPDMs), assessed by western blot. GAPDH was used as loading control. (**C**) H4 cells were pre-treated with PDMs for 16 h, then subjected to 600 µM of H_2_O_2_ for 6 h. Viability was assessed by PI fluorescence *versus* side scatter (SSC), determined by flow cytometry. PI positive cells are gated, and the respective percentage is presented, statistics were performed in comparison to the control. (**D**) Mitochondrial membrane potential evaluated using MitoTracker Red by flow cytometry; histogram of MitoTracker Red fluorescence (left panel) and respective median fluorescence intensity (MFI) (right panel) of H4 cells transfected with the empty vector (control), co-transfected with SynT and Synphilin-1 not treated (SynT) or treated with LPDMs (SynT + LPDMs). (**E**) H4 cells transfected with the empty vector (control), co-transfected with SynT and Synphilin-1 (SynT) or treated with LPDMs (SynT + LPDMs) were immunostained for SynT (green). Mitochondria were visualized using MitoTracker Red (red) while Nuclei were stained with DAPI (blue). Scale bar, 10 µm. A representative result is shown, and values represent the mean ± SD of at least three independent experiments. Statistically significant differences between the indicated treatments are shown, *p < 0.05, **p < 0.01, ***p < 0.001. Full-length immunoblots are shown in Supplementary Figure S4.

Next, we investigated the effect of the LPDMs on aSyn aggregation in human cells (Fig. 5A, left panel)^48,49^. Twenty-four hours after co-transfection of cells with plasmids encoding for SynT and Synphilin-1, cells were treated with LPDMs for 16 h. We observed that LPDMs treatment increased the percentage of cells without aSyn inclusions and reduced the percentage of cells presenting more than 10 inclusions (Fig. 5A, middle panel). The mean fluorescence intensity (MFI) of the inclusions in the microscopy images was assessed in cells treated with the LPDMs or vehicle, we did not observe a statistical difference in inclusions MFI between treatments. Importantly, the effect observed was not due to differences in SynT levels due to LPDMs treatment, evaluated by immunoblotting (Fig. 5B).

The effect of the LPDMs on oxidative injury was analyzed in human H4 cells treated with H_2_O_2_ (Fig. 5C). We observed that the pre-treatment with LPDMs for 16 h, prior to stress with 600 µM H_2_O_2_ for 6 h, protected cells from oxidative stress-induced cell death, as observed by the reduction of the number of PI positive cells (gated cells) (Fig. 5C). These data show that the LPDMs protected human cells from oxidative stress, validating the results obtained in yeast.

The mitochondria work as generators and targets of ROS^13^ and its function is impaired by aSyn^32,33^. Mitochondrial function was studied using MitoTracker Red, a dye that stains mitochondria in live cells and whose accumulation is dependent on mitochondrial membrane potential, a parameter linked to mitochondrial function. In cells expressing SynT, MitoTracker Red fluorescence was lower than in control cells, indicating reduced mitochondrial membrane potential and suggesting impairment of mitochondrial function. On the other hand, treatment with LPDMs of cells expressing SynT increased the MitoTracker Red signal, suggesting a rescue of the mitochondrial function (Fig. 5D). The observation of mitochondria by fluorescence microscopy reinforces the FCM data (Fig. 5E).

## Discussion

PD is currently an incurable disorder affecting a growing number of people due to the aging of population^11^. Therefore, it is urgent to understand the molecular mechanisms underlying PD pathogenesis and to identify novel therapeutic targets and strategies. Phenolic compounds, in food, isolated, or in extracts, are of great interest in nutrition and medicine. They have been associated with the prevention of cancer, chronic and neurodegenerative diseases^1,3–6^. Thus, (poly)phenols are attractive compounds in the context of neurodegenerative diseases, since public health policies are focused on the prevention of dementia, through the promotion of a healthy life style, natural nutrition and supplementation^1^.

In this pioneer study of digested metabolites in PD, we found that PDMs from *A. unedo* leaves offered significant cytoprotection in established yeast and human cell models of synucleinopathies^19,29,48,49^. *A. unedo* possesses several useful biological properties^3,27,28,50^, nevertheless its neuroprotective potential was not explored. After digestion, the metabolites obtained consisted mainly of myricetin-3-rhamnoside, quercetin-hexoside and kaempferol glycosides. The latter was described as being neuroprotector against ischemic brain injury and neuroinflammation in rats^51^, while, myricetin and quercetin were described to efficiently inhibit aSyn fibrillation^52^.

Interestingly, in a previous study we observed that the original undigested fraction of *A. unedo* leaves was not protective against aSyn-induced toxicity in the conditions tested, measured by growth curves and cells metabolic capacity^24^. Therefore, the results here obtained indicate that the digestion potentiates the beneficial effects of (poly)phenols in *A. unedo*, reinforcing their nutritional relevance. Importantly, digestion was also found to potentiate neuroprotective properties of blackberry (poly)phenols^53^.

PD pathogenesis is associated with proteostasis dysfunction and inefficient protein clearance^12,21^. Autophagy was shown to be required for the degradation of aSyn aggregates under pathological conditions^21,24,32,37,38^. Nevertheless, recent studies reported that increased autophagy/mitophagy, promoted by aSyn, has a deleterious effect in aged cells^32,43^. This appears counterintuitive, as autophagy is generally considered to be a pro-survival process in protein misfolding diseases^21^. However, autophagy is a complex pathway and, depending on the conditions and models used, it can be either a survival or death mechanism. In the conditions tested herein, aSyn induces the activation of autophagy, as measured by Atg8 protein and mRNA levels. However, this autophagy is dysfunctional, as observed by the reduced autophagic flux. Ultimately, the excessive activation of a dysfunctional autophagy will lead to a loss of selectivity, resulting in the trapping of functional competent organelles in autophagosomes, contributing to the toxicity observed. In fact, LPDMs reduced aSyn toxicity and aggregation, and improved autophagic function, as observed by the increased autophagic flux, in agreement with our previous work where we found that (poly)phenols preferentially affect the autophagic function^24^, which is impaired by aSyn expression. Nevertheless, it is not clear if the effect of LPDMs on the quality control pathways is direct or a consequence of the modulation of aSyn aggregation.

**Figure 6:**
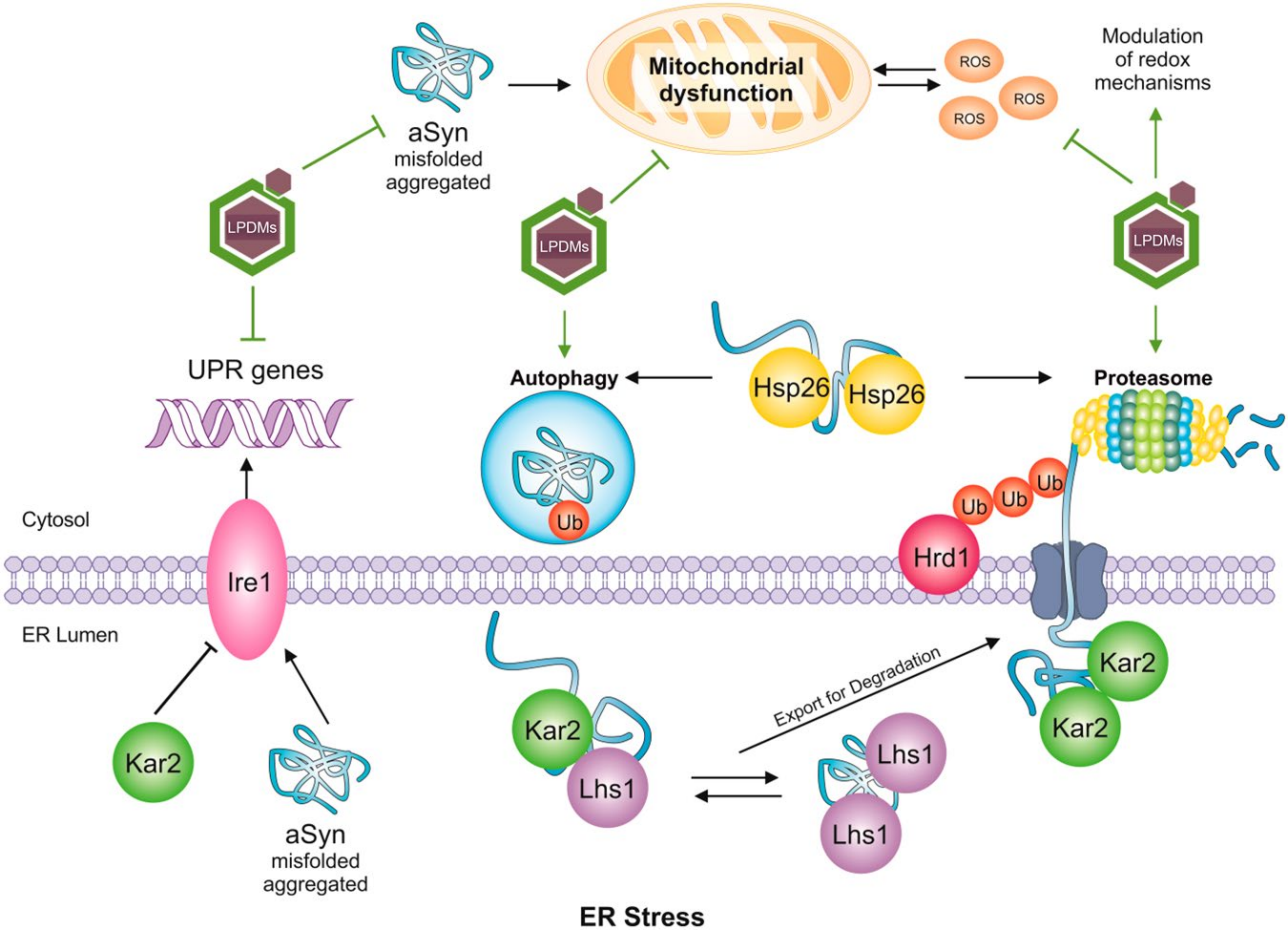
Schematic representation of the mechanism of aSyn toxicity and protection by *A. unedo* LPDMs. Accumulation of unfolded proteins (such as aSyn) or oxidative stress leads to endoplasmic reticulum (ER) stress with activation of the unfolded protein response (UPR), leading to oligomerization of the kinase Ire1, and transcription of genes related with protein-folding homeostasis. Kar2 is a negative regulator of UPR via interaction with Ire1. When unfolded proteins accumulate Kar2 is displaced from Ire1, to act as a chaperone aiding proteins conformation. Direct activation of Ire1 by unfolded proteins is an alternative mode for UPR induction. Lhs1, an ER chaperone, mediates protein folding and interacts with Kar2. Cytosolic chaperones also assist aSyn conformation, as Hsp26. Proteins failing to be correctly folded are exported from the ER, a process involving Kar2. Hrd1 is an ubiquitin ligase responsible for recognizing and ubiquitinating misfolded proteins. Afterward ubiquitination, proteins are either degraded in the cytosol by the proteasome or by autophagy, pathways that are both upregulated by the UPR. Aggregated aSyn induces reactive oxygen species (ROS) production and elicits ER stress, with concomitant induction of UPR gene expression. Our findings showed that LPDMs reduced aSyn aggregation and the induction of UPR, while promoting autophagy and proteasome function, pathways involved in aSyn degradation. Moreover, LPDMs reduced oxidative stress and mitochondrial dysfunction, by modulation of redox homeostasis. Overall, LPDMs increased cell viability by a pleiotropic protective role.

To ascertain the pathways/molecules associated with aSyn-induced autophagy activation, we investigated the UPR pathway and the levels of ROS. We analysed the expression of ER stress associated genes, where Kar2 and Lhs1 are HSP70 molecular chaperones found in the ER, where they mediate protein folding^54,55^. Kar2 negatively regulates the UPR via interaction with Ire1^14,54^, a major regulator of the UPR. Lhs1 acts as a nucleotide exchange factor for Kar2 and is upregulated by the UPR^55^. Hsp26 is a cytosolic chaperone that binds and prevents unfolded proteins from irreversibly forming large protein aggregates^56^. Hsp26 activity is found only under stress conditions, where it is strongly induced^56^. Hrd1 is an ubiquitin ligase responsible for recognizing and ubiquitinating misfolded proteins in the ER, for further degradation by the proteasome and is induced in UPR conditions^57^ (Fig. 6). UPS and autophagy are both mitigators of ER stress^16,17,42^. Accordingly, increasing evidence suggests the existence of a complex cross-talk between different proteostasis pathways, processes known to play distinct roles in the clearance of specific species of aSyn^18,22,23,37^.

Our findings demonstrate that aSyn-elicited ER stress and the concomitant activation of the UPR, might underlie the observed induction of genes related with autophagy and proteasomal function. LPDMs reduced both autophagy activation and the induction of UPR genes, while promoting autophagy and proteasome function (Fig. 6). In line with this observation, it is known that Rpn4, a transcription factor that stimulates the expression of proteasome genes, is regulated by the 26S proteasome in a negative feedback control mechanism^41^.

The reduction in the levels of *LHS1* and *RPN4* mRNA by LPDMs is in agreement with an extensive screen for small molecules capable of attenuating aSyn toxicity^58^. In this study, aSyn expression induced the upregulation of *LHS1* and *RPN4*, among other genes, and the protective compounds markedly reversed the transcriptional changes induced by high levels of aSyn^58^, as observed here for the LPDMs under study.

ROS, particularly superoxide anions, are crucial signaling molecules implicated in the control and regulation of autophagy and aging^59^. Sod1 and Sod2 are superoxide dismutase proteins responsible for detoxifying superoxide radical, which are tightly regulated at transcription level in response to oxidative stress^60^. Sod1 is mainly cytosolic while Sod2 is found in the mitochondria, both are strongly implicated in the aging of yeast cells, and their deletion dramatically reduces the chronological and replicative life span of yeast^59^. Moreover, the efficiency of the protein folding status of the ER depends on specific redox condition in its lumen^14^. Our data suggest that the upregulation of *SOD1* and *SOD2* might be the underlying mechanism behind LPDMs-mediated decreased in superoxide anion levels, a ROS involved in mitochondrial functionality^13^. These observations are further substantiated by recent studies of the activity of antioxidant enzymes in PD models. Induction of PD by the expression of mutant aSyn^61^ or DJ-1^62^ in flies, and MPP^+^ exposure in a human cell line^63^, resulted in reduced SOD activity and increased production of superoxide. It was proposed that the increase in ROS levels observed in the PD models was due to a deficient activity of the antioxidant enzymes. In addition, treatment with protective compounds increased SOD activity and rescued the PD phenotype^61–63^. Moreover, treatment with (poly)phenols reduced the ratio of oxidized/reduced glutathione (GSSG/GSH), supporting their role against oxidative stress damage. In our study, the evidence that LPDMs improved oxidative homeostasis and mitochondrial function was further confirmed in human cells, as treatment with LPDMs increased mitochondrial membrane potential in cells expressing aSyn.

Mammalian SIRT1 was recently described as an inducer of autophagy under starvation conditions, and is required to sustain autophagy through deacetylation of autophagic regulators, including Atg5, Atg7, Atg8 and Atg12^64^. Here, we also investigated the effect of LPDMs on Sir2, the yeast orthologous of SIRT1. Notably, *SIR2* overexpression increases aSyn toxicity by regulating *ATG8* and, particularly, *ATG32* mRNA, leading to excessive mitophagy^43^. Our findings revealed that aSyn expression induced *SIR2* and, impressively, LPDMs treatment maintained *SIR2* expression at basal levels, in line with the autophagy induction results, and the described effect of acetylation on autophagy regulation^64^. Altogether, the effect of LPDMs on *SIR2* expression, autophagy flux and aSyn toxicity protection are in agreement with previous studies where it was observed that deletion of the *SIR2* gene alleviated aSyn toxicity and drastically inhibited autophagy induction^43^.

Overall, LPDMs reduced aSyn toxicity and aggregation, promoting the formation of smaller oligomeric species. LPDMs also enhanced the efficiency of the UPS and autophagy and reduced oxidative stress. In total, our study provides important hints for the development of nutraceutical/therapeutic strategies against PD and other synucleinopathies.

## Materials and Methods

### Plant material and extraction procedure

Fruits and leaves of *A. unedo* L. were collected by random sampling in an extensive area of Arrábida Natural Park. Plant material was harvested, frozen, freeze-dried and ground in an IKA M20 mill to pass a 0.5 mm sieve and stored at −80 °C. Extracts were prepared using 1 g of lyophilized powder to 12 mL of 50% (v/v) hydroethanolic solution as described^53^.

### *In vitro* digestion

*In vitro* digestion was performed as previously described^25^. Briefly, pH of the extract was adjusted to 1.7. Afterwards 315 units.mL^−1^ of porcine pepsin (Sigma ref P6887) was added and the solution was incubated at 37 °C, 2 h, 100 rpm. Afterwards 4 mg.mL^−1^ pancreatin from porcine pancreas (Sigma ref P7545) and 25 mg.mL^−1^ bile salts mixture were added. A segment of cellulose dialysis tubing (cut-off 12 kDa) containing 0.1 M NaHCO_3_ to neutralize titratable acidity, was placed inside the beaker containing the solution. After 2 h at 37 °C, the solution inside and outside the dialysis tubing were collected. The soluble materials were applied to C18 solid phase extraction columns (GIGA tubes, 1000 mg capacity, Phenomenex Ltd.), as described in^53,65^. The (poly)phenol-digested metabolites (PDMs) obtained were concentrated and freeze-dried to suitable phenol concentrations determined by the Folin-Ciocalteau method^53^.

### Liquid chromatography mass spectrometric (LC-MS) analysis of phenolic profile

The samples were dissolved in 1.25 mL water/acetonitrile (80:20, v/v) and 0.4 mL was transferred to a 0.45 mm filter vial. Samples were injected via an autosampler onto a Hypersil Gold (50 mm × 2.1 mm; 1.9 µm, Thermo Scientific) reverse-phase UPLC column at 30 °C. The HPLC system consisted of an Accella 600 quaternary pump and an Accella PDA detector, coupled to an LTQ Orbitrap mass spectrometer (Thermo Fisher Scientific, Stafford House, Boundary Way, Hemel Hempstead, UK). The mobile phases were solution A (0.1% (v/v) aqueous formic acid) and solution B (0.1% (v/v) formic acid in acetonitrile/water (50:50, v/v). The flow rate was 450 µL min^−1^, and the gradient was 0 min; 97% A, 0–3 min; 85% A, 3–7 min; 75% A, 7–10 min; 50% A, 10–13 min; 50% A, 13–14 min 0% A, 14–16 min 0% A, 16–17 min 97% A, 17–20 min 97% A). The PDA detector range was 200–600 nm. Mass spectrometer mass range was *m/z* 80–2000 with alternative full scan MS and MS/MS data-dependent scans in negative mode^66^. Exact mass data was used to predict the molecular formula using the resident software Xcalibur™ (errors <2 ppm). Components were identified using PDA maxima, *m/z* and MS^2^ high-resolution fragmentation data with study of the suggested formula for the fragments, and comparison with the values available in the databases.

### Liquid chromatography UPLC-MS-NMR analysis of (poly)phenolic profile

The samples were dissolved in 200 μL methanol, were filtered with a 0.22 µm filter and transferred to HPLC vials. Samples were injected via an autosampler reverse-phase UPLC column at 30 °C. The UPLC system consisted of a Waters Acquity UPLC with a BEH C18 Waters (2.1 × 50 mm, 1.7 µm) and a Quadrupole–TOF mass spectrometer (QTOF maXis impact, Bruker, Daltonics, Bremen, Germany), using an orthogonal Z-spray-ESI interface operating in negative ion mode detector, coupled to an NMR. This setup was coupled to a Bruker Avance III HD spectrometer equipped with Ascend TM magnet, 11.7 T (1H operating frequency 500 MHz) and a cryoprobe (Cryoplatform Prodigy BBO) for increased sensitivity. The mobile phases used were solution A (0.1% (v/v) aqueous formic acid) and solution B (0.1% (v/v) formic acid in acetonitrile/water (50:50, v/v)). The flow rate was 5 µL min-1, and the gradient was 0 min; 97% A, 0–3 min; 85% A, 3–7 min; 75% A, 7–10 min; 50% A, 10–13 min; 50% A, 13–14 min 0% A, 14–16 min 0% A, 16–17 min 97% A, 17–20 min 97% A. The PDA detector range was 280–350 nm. Mass spectrometer mass range was m/z 80–2000 with alternative full scan MS and MS/MS data-dependent scans in negative mode. ^1^H NMR spectra were obtained at 500 MHz in CD3OD with chemical shift values (δ) in ppm.

### Yeast transformation and plasmids

BY4741 (*MAT*a; *his3Δ1*; *leu2Δ0*; *met15Δ0*; *ura3Δ0*) cells were transformed using lithium acetate standard method^67^. The empty plasmids pRS426GAL, p413-GPD or p415 were used as control^68^. The pRS316-GFP-*ATG8* plasmid was a gift from Prof Yoshinori Oshumi (National Institute for Basic Biology, Okazaki, Japan) and was used to sub-clone GFP-*ATG8* as well as the endogenous *ATG8* promoter into the *SacI* - *XhoI* sites of p415 plasmid^39^. Other plasmids used were previously described: p426GAL-aSyn-GFP and p426GAL-aSyn carrying the human gene of aSyn with or without a C-terminal fusion to GFP, under the regulation of *GAL1* inducible promoter^19^; p413-GPD-GFPu expressing an unstable GFP under the regulation of *GPD* constitutive promoter^19^.

### Yeast growth and compound testing

Cells in *log* growth phase were obtained using synthetic complete (SC) medium [0.67% (w/v) yeast nitrogen base without amino acids (Difco), 1% (w/v) raffinose and the appropriate auxotrophy supplement mixture (QBiogene)], 200 rpm at 30 °C^24^. aSyn expression was induced by growing cells (OD_600 nm_ 0.2) in SC selective medium 1% (w/v) galactose (aSyn ON) not supplemented or with 62 μg GAE.mL^−1^ leaf or fruit PDMs, for 6 h at 30 °C, 200 rpm. For spot assays D_600 nm_ of treated cells was set to 0.1 ± 0.005 and 1:10 serially dilutions were prepared^24^. Then, 4 µL of each dilution was spotted in solid SC solid selective medium 1% (w/v) galactose and incubated at 30 °C, 42 h. Images were acquired using Chemidoc^TM^ XRS and Image Lab software. For clearance experiments, after 6 h of aSyn expression induction (with or without LPDMs treatment), cells were centrifuged, washed in PBS, resuspended in 2% (w/v) glucose SC liquid medium (aSyn expression OFF) and incubated at 30 °C, 200 rpm, 18 h. aSyn levels were determined by western blotting at 6 h of induction (corresponding to 0 h of clearance) and 18 h of clearance.

### H4 cell culture and transfections

Human H4 neuroglioma cells (gift from Dr. Bradley T. Hyman, Harvard Medical School) were maintained at 37 °C in OPTI-MEM I (Gibco, Invitrogen, Barcelona, Spain) supplemented with 10% (v/v) fetal bovine serum and seeded at 80,000 cells.cm^−2^ density 24 h prior to transfection. Cells were transfected as previously described with pcDNA3.1-aSynT and pcDNA3.1-Synphilin-1^48,49^. For H_2_O_2_ toxicity assay cells were treated with 2 μg GAE. mL^−1^ *A. unedo* LPDMs for 16 h, the medium was removed and cells were treated with 600 µM H_2_O_2_ for 6 h, viability was assessed using PI and the percentage of cells labeled with PI is presented (gated cells). To evaluate the effect of LPDMs in aSyn aggregation, 24 h after transfection cells were treated with 2 μg GAE. mL^−1^ LPDMs for 16 h.

### Fluorescence microscopy

To determine the percentage of yeast cells with aSyn inclusions, cells were grown as described above and GFP fluorescence was visualized, the percentage of cells presenting aSyn inclusions was determined by counting at least 800 cells for each treatment. Transfected H4 cells were fixed and permeabilized with methanol and blocked in 1.5% (v/v) normal goat serum in PBS for 1 h. Cells were incubated with primary antibody overnight at 4 °C (mouse anti-aSyn; BD Transduction Laboratories, San Jose, CA, USA) followed by secondary antibody incubation for 1 h (goat anti-mouse IgG-Alexa488, Invitrogen Corporation, Carlsbad, CA, USA). For mitochondria assays, cells were labeled with 200 nM MitoTracker Red FM (Invitrogen Corporation, Carlsbad, CA, USA), for 30 min at 37 °C protected from light. To stain the nucleus 4 µg. mL^−1^ of DAPI (Sigma-Aldrich, St. Louis, MO, USA) was used. The proportion of cells with aSyn inclusions within the population was then determined by counting at least 100 cells per condition. Slides were subjected to fluorescence microscopy with a Zeiss Axiovert 200 M Widefield Fluorescence microscope and the counting was performed using ImageJ software.

### Western blotting

For aSyn and GFP-Atg8 quantification total yeast protein extraction was performed using the method described^69^. Atg8 induction was quantified by the determination of the fold increase of total GFP signal (GFP-Atg8 and free GFP signal, detected with anti-GFP) normalized to PGK; autophagic flux was quantified by measuring the vacuolar degradation of the Atg8 domain reporter (ratio of free GFP to total GFP signal)^40^. For aSyn quantification in H4 cells, cells were lysed with NP-40 lysis buffer in the presence of protease and phosphatase inhibitor cocktail (Roche, Mannheim, Germany), western blot was performed following standard procedures. Antibodies used: aSyn (BD Transduction Laboratories, San Jose, CA, USA), GAPDH (Ambion, Cambridgeshire, UK), GFP (Antibodies Incorporated, Davis, CA, USA), PGK (Life Technologies, Paisley, UK). The western blots shown are representative of three independent experiments with similar outcomes, densitometry is measured from the western blot panel shown. The protein bands were quantified using ImageJ software for statistical analysis.

### Sucrose gradients

Total protein was obtained from cells expressing aSyn and applied on a 5 to 30% (w/v) sucrose gradient as described before^37^. Fractions were collected, precipitated for 4 h at 4 °C in trichloroacetic acid, washed in acetone three times and suspended in protein sample buffer (0.5 M Tris-HCl, pH 6.8, 10% (w/v) glycerol, 0.1% (w/v) SDS, 6 mM bromophenol blue). Proteins were resolved by SDS-PAGE, the estimation of the molecular sizes for each fraction was previously described^70^. The western blots shown are representative of three independent experiments with similar outcomes, densitometry is measured from the western blot panel shown. The protein bands were quantified using ImageJ software for statistical analysis.

### Flow cytometry (FCM)

FCM was performed in a FACS BD LSR Fortessa, equipped with the 695/40 BP and the 685 LP, as previously described^24^. ROS was determined in yeast cells transformed with the aSyn encoding plasmid or the empty plasmid, incubated with 30 µM DHE (dihydroethidium), for 15 min at 30 °C, 200 rpm. For cell viability cells transformed with the aSyn-GFP encoding plasmid or the empty plasmid were incubated with PI 5 µg.mL^−1^, for 15 min at 30 °C, 200 rpm. To study the proteasome, yeast cells were transformed with the aSyn and GFPu encoding plasmid or the respective empty plasmid. For mitochondria assays in H4 cells, cells were labeled with 200 nM MitoTracker Red FM, for 30 min at 37 °C. Data analysis was performed using FlowJo software. A minimum of 10000 events were collected for each experiment. Results were expressed as median fluorescence intensity (MFI) of a molecule. Image J was used for cropping and quantification of bands.

### Real-time PCR

Quantitative real-time PCR (qRT-PCR) analyzes was performed as described in^43^. The oligonucleotides listed in Supplementary Table 1 were used to evaluate expression of *SOD1*, *SOD2*, *HAP4*, *LHS1*, *KAR2*, *HRD1*, *GSH1*, *GLR1*, *RPN4*, *SIR2*, and *ATG8* genes. Relative standard curves were constructed for each gene, using triplicate serial dilutions of cDNA. The relative expression of the genes was calculated by the relative quantification method with efficiency correction, using Applied Biosystems^®^ ViiA™ 7 software. *ACT1*, *PDA1* and *PGK* were used as internal standards and for the normalization of mRNA expression levels^43^. The results were expressed as fold change mRNA levels relative to the control condition and normalized to the reference genes (relative expression).

### Glutathione (GSH) and glutathione disulphide (GSSG) quantification

Yeast cells were grown as previously described and GSH and GSSG were quantified by HPLC after sample derivatization, as described^65^.

### Statistical analysis

The results reported in this work are the average of at least three independent biological replicates and are represented as the mean ± SD. Differences among treatments were assessed by analysis of variance with Tuckey HSD (Honestly Significant Difference) multiple comparison test (a = 0.05) using SigmaStat 3.10.

### Data availability

The datasets generated during and/or analyzed during the current study are available from the corresponding author on reasonable request.

## Electronic supplementary material

Supplementary information
