# Supplementary material for: (Poly)phenol-digested metabolites modulate alpha-synuclein toxicity by regulating proteostasis
Source: Sci Rep. 2018 May 3;8:6965. doi: 10.1038/s41598-018-25118-z (PMC5934470; doi:10.1038/s41598-018-25118-z)
Supplement: Supplementary file 1 — Supplementary information [file 41598_2018_25118_MOESM1_ESM.pdf]

(Poly)phenol-digested metabolites modulate alpha-synuclein toxicity by regulating proteostasis

Diana Macedo, Carolina Jardim, Inês Figueira, A. Filipa Almeida, Gordon J. McDougall, Derek Stewart, Jose, E. Yuste, Francisco A. Tomás Barberán, Sandra Tenreiro, Tiago F. Outeiro and Cláudia N. Santos

Supplementary Table 1

Gene		Sequence	Eff (%)
GLR1	F	CGACCGGTGGAAAGGCTATT	106
	R	CAGCGCCAACAACAACAACT	
RPN4	F	GCTTCGATACCCCCACAACA	107
	R	TCTATCGTTGGCCGTTGCTT	
HAP4	F	TTCTACTACAGGCCTCCGCT	104
	R	TGGTTGGTATTTGGGGCGAT	
ATG8	F	AATATCTAGTTCCTGCTGACC	108
	R	CCGTCCTTATCCTTGTGTT	
SIR2	F	CGAACTTCCACTATGCCCGT	100
	R	GGAGGCCTTTCCGACATTGA	
KAR2	F	GTCCAAGCCACTTCTGGTGA	100
	R	CATTTGGCTGGACAAGGCAC	
LHS1	F	AGGGTCTTTAGCAGCCGTTT	100
	R	TTTTGCCTCTGGTGTGAGCA	
SOD1	F	CGAGCCAACCACTGTCTCTT	97
	R	ACGTTACCCATGTCACCGAC	
SOD2	F	ACATCAAGTTCCATGGCGGT	104
	R	CTGCTCGTCGATTGCCTTTG	
GSH1	F	ATGGGCTGTTCGTGCTTACA	112
	R	AACGAACATCTTGGTCGGCT	
HRD1	F	AACAGCTCGACGACACTCTTGTCA	85
	R	TCCACGTCTGCTGGTTTGGAGAAT	
HSP26	F	CTACGCACCAAGACGTCAGT	100
	R	ACCAGATGGGAACAGGGACA	
ALG9	F	CGGGAAGCTTGCTCCTGTAT	82
	R	CTAGCACAGGCAGTGGGAAA	
PGK	F	GTGCCAAGGTTGCTGACAAG	72
	R	GAACGATTTCAGCACCAGCC	
PDA1	F	CTGTTGGTCAGGAGGCCATT	65
	R	GCATGGAACCACCCTTACCA	

Supplementary Figure 1

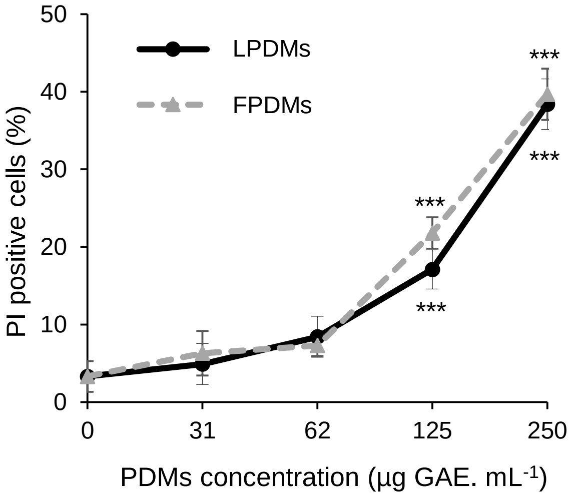

FIGURE S1. *A. unedo* LPDMs and FPDMs toxicity in control cells. Yeast cells were treated with the indicated concentrations of PDMs for 6 h, in galactose liquid medium and cell viability was assessed by flow cytometry using propidium iodide (PI). Values represent the mean ± SD of three independent experiments. Statistically significant differences between the indicated treatments and cells not treated with PDMs are shown, ***p<0.001.

Supplementary Figure 2

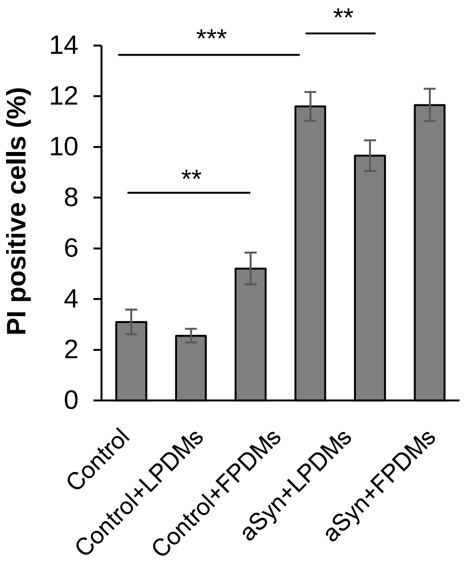

FIGURE S2. *A. unedo* LPDMs reduce aSyn-induced toxicity. Yeast cells transformed with a plasmid encoding for aSyn or with the empty vector (control), were treated with 62 μg GAE.mL^{-1} of LPDMs or FPDMs for 6 h, in galactose liquid medium. Cell viability was assessed by flow cytometry using propidium iodide (PI). Values represent the mean ± SD of three independent experiments. Statistically significant differences between the indicated treatments are shown, **$p<0.01$, ***$p<0.001$.

Supplementary Figure 3

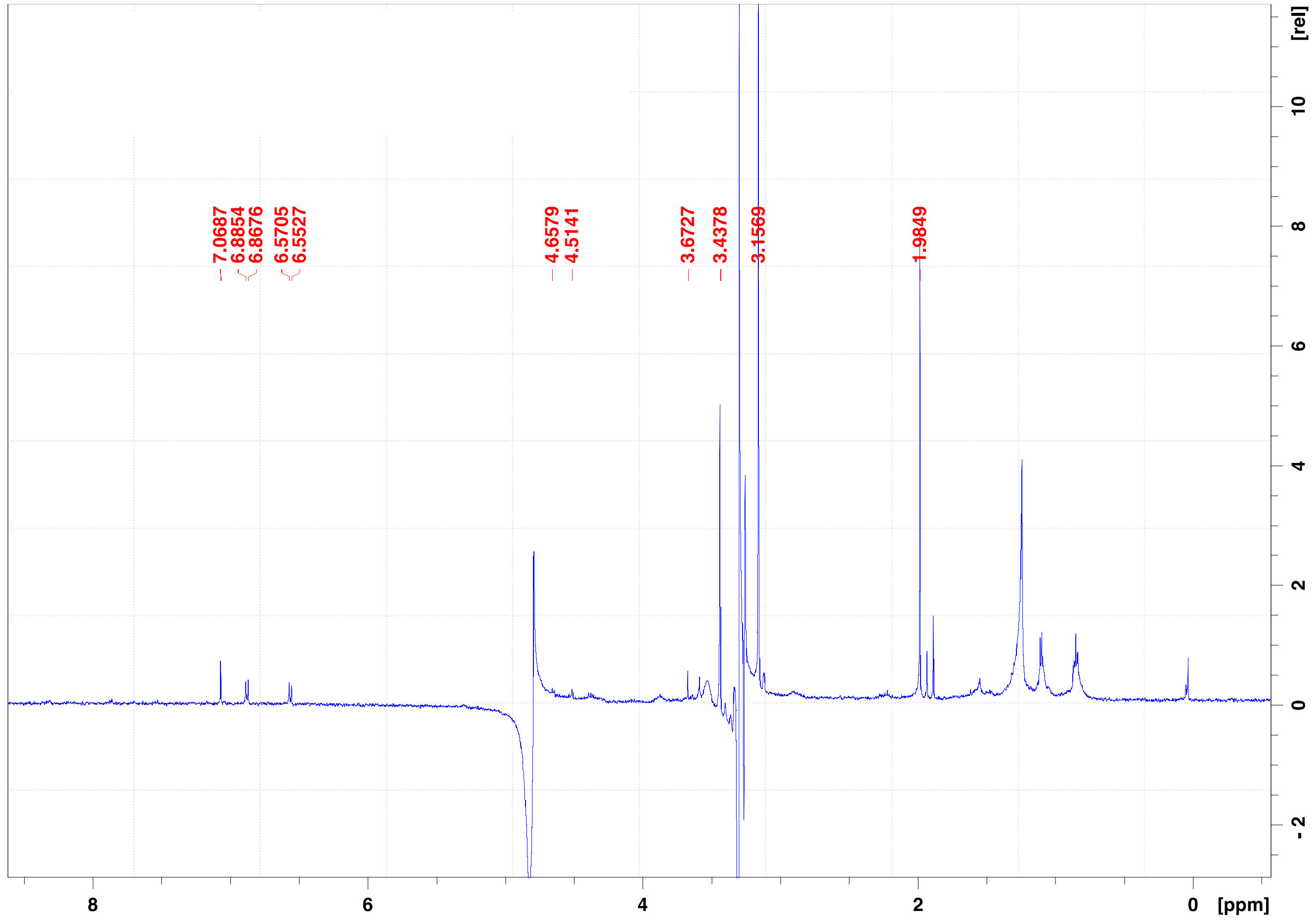

FIGURE S3. ^{1}H-NMR of arbutin gallic acid ester with *m/z* 423.

Supplementary Figure 4

A

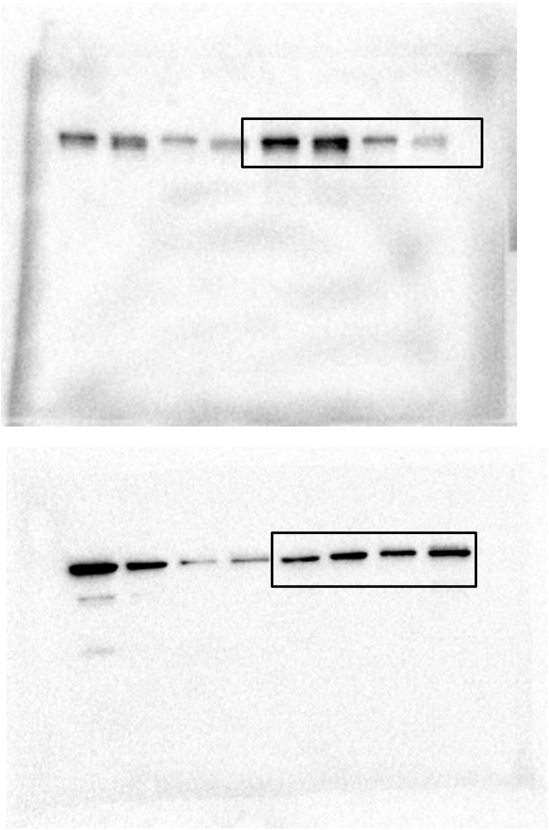

C

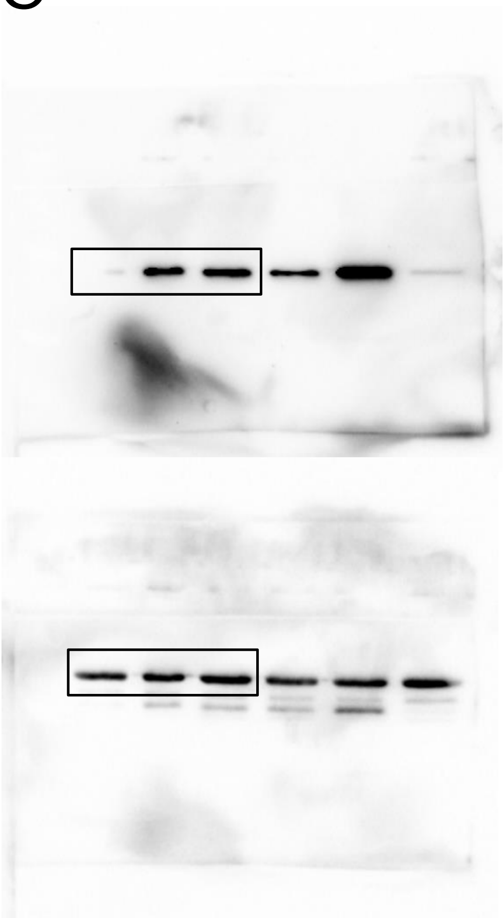

B

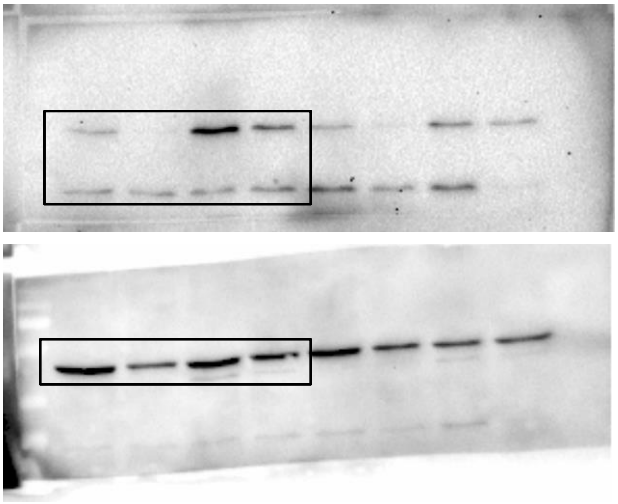

FIGURE S4: Uncropped gels for figures **A)** 3A, **B)** 3C and **C)** 5B.
